# Estimating Snow-Related Daily Change Events in the Canadian Winter Season: A Deep Learning-Based Approach

**DOI:** 10.3390/jimaging11070239

**Published:** 2025-07-14

**Authors:** Karim Malik, Isteyak Isteyak, Colin Robertson

**Affiliations:** 1 School of the Environment, University of Windsor, Windsor, ON N9B 3P4, Canada; isteyak@uwindsor.ca; 2 Department of Geography and Environmental Studies, Wilfrid Laurier University, Waterloo, ON N2L 3C5, Canada; crobertson@wlu.ca

**Keywords:** Siamese Attention Network, snow water equivalent, daily snow variability, structural similarity, snow-related change events, climate change

## Abstract

Snow water equivalent (SWE), an essential parameter of snow, is largely studied to understand the impact of climate regime effects on snowmelt patterns. This study developed a Siamese Attention U-Net (Si-Att-UNet) model to detect daily change events in the winter season. The daily SWE change event detection task is treated as an image content comparison problem in which the Si-Att-UNet compares a pair of SWE maps sampled at two temporal windows. The model detected SWE similarity and dissimilarity with an F1 score of 99.3% at a 50% confidence threshold. The change events were derived from the model’s prediction of SWE similarity using the 50% threshold. Daily SWE change events increased between 1979 and 2018. However, the SWE change events were significant in March and April, with a positive Mann–Kendall test statistic (*tau* = 0.25 and 0.38, respectively). The highest frequency of zero-change events occurred in February. A comparison of the SWE change events and mean change segments with those of the northern hemisphere’s climate anomalies revealed that low temperature and low precipitation anomalies reduced the frequency of SWE change events. The findings highlight the influence of climate variables on daily changes in snow-related water storage in March and April.

## 1. Introduction

Snow is an essential landcover type in the cold regions and plays a critical role in the Earth’s hydrology, surface energy balance, and climate regime variability. It is estimated that approximately 65% of the Canadian landmass is covered with snow during the winter [1]. Snowmelt produces water that feeds freshwater ecosystems and contributes to irrigation [2]. Snow cover, in combination with ice melt deposition in the ocean, further influences the sea energy budget and ice mass balance, as well as aquatic species’ productivity [3]. Snowfall also modulates sea ice thermodynamics by forming thicker snowpacks that reduce heat loss [4].

Snow stratigraphy and physical characteristics respond naturally to environmental variables. However, climate warming is likely to exacerbate changes in snow parameters with unpredictable impacts on humans and ecosystems. Rapid daily change in the snowmelt process, primarily driven by human-induced climate warming, could trigger catastrophic events such as floods, landslides, and hydropower system collapse [5]. For example, snowmelt-driven avalanches impact the safety of humans and infrastructure [6,7]. Additionally, earlier snowmelt off alters the timing and volume of snow-related runoff, increasing the occurrence of spring flooding and summer droughts [8,9,10]. Soil temperature, water availability, and soil microbial respiration are also affected by snow dynamics [11,12,13]. Furthermore, early snowmelt has deleterious consequences on leisure activities such as over-snow vehicle recreation and skating [14,15].

Snow water equivalent (SWE)—the amount of water that will be yielded if a given snowpack melts, snow cover extent (SCE)—the area of ground covered by snow, and snow depth (SD)—the vertical depth of ground snow, are highly sensitive snow parameters and thus sentinels of climate change. These snow parameters have been extensively used to investigate the effects of climate change and global warming in the Arctic and other cold regions. Significant progress has been made toward characterizing trends in SWE, snow depth, and snow density [16], evaluating SCE and snow properties [17]. For instance, analysis of gridded snow data has shown declining SWE trends in Eastern and Western Canada in March [18]. Similarly, Pulliainen et al. [16] used GlobSnow’s v3.0 dataset to illustrate that snow mass is declining across North America in March. In a related study, SCE was shown to be declining in the northern hemisphere [19]. However, as the ensemble of available SWE data is derived from varying inputs, an accurate understanding of snow variability, climatology, and historical trends would require comparing the ensemble of available data products [20].

Despite the progress in snow trend studies, snow is not a consistent sentinel of climate-forcing; snow is influenced by diverse variables, including the snow-albedo feedback [21,22,23]. Furthermore, the relationship between seasonal snow and climate variables is uncertain and non-linear [24]. For example, snow cover response to temperature and precipitation varies with latitude, whereas excessive precipitation biases the estimates of snow distribution [25,26]. In many studies, snow trend analysis is based on selecting reference periods and averaging time series snow observations [27]. Also, snow data in March is frequently used to analyze SWE trends [28]. Comparative analysis of snow trends during all the winter months will provide valuable information on snow parameter response to climate variables.

Quantifying changes in snow parameters at high temporal resolution is an effective method for inferring the effects of global climate trends on snow dynamics during the winter season [29]. Spatial–temporal patterns of daily SWE melt, however, are largely understudied, partly due to low signal-to-noise ratio in daily SWE, uncertainty in gridded SWE data products, and the tendency for algorithms to detect internal variability in SWE that is not directly related to the underlying climate variables [25,30]. Furthermore, given that detecting and attributing changes in snow is not a straightforward task [26], methods invariant to stochastic internal variability and seasonal patterns of snow spatial structure are crucial for obtaining accurate estimates of changes in snow parameters.

Computer vision and deep learning methods have excelled at pattern recognition involving the comparison of spatial and temporal variability in Earth Observation data [31]. The U-Net, a fully convolutional CNN, is one such deep learning model [32]. To overcome the limitations of the classical U-Net, sophisticated variants such as the residual U-Net, Dense U-Net, and the Attention U-Net were developed [33,34]. Multiscale attention transformers have been introduced to detect change [35,36,37]. The fully convolutional feature extraction, skip or residual connections, and an encoder–decoder module in the U-Net ensure the models retain spatial information that characterizes local image structures.

This study builds on Malik and Robertson’s [31] previous work on change detection in SWE using the U-Net model. In the wake of climate change, monitoring snow processes at daily temporal resolution is becoming more imperative to improving human well-being and economic viability, as well as predicting, managing critical infrastructure, and adapting to snow-related disasters. Therefore, the overarching objectives of this study are to (a) demonstrate Siamese Attention U-Net’s capability to detect daily similarity and variability in SWE, (b) estimate the frequency of daily SWE change events during winter, and (c) link SWE change events to climate anomalies.

## 2. Related Work

### 2.1. Progress in Snow Data Acquisition

Snow data collection began with in situ weather stations, which remain operational to date. Snow Telemetry (SNOTEL) and Canada’s SWE (CanSWE) are typical point-wise in situ snow data derived using a constellation of stations across the conterminous United States and Canada, respectively [38]. These datasets have proved vital for snow parameter studies [18]. Point-wise snow measurements, however, tend to lack complete spatial continuity. To address this inherent discontinuity, gridded snow data products have been developed using in situ data combined with spatial interpolation, machine learning, passive microwave, and real-analysis techniques [39,40,41].

Gridded snow data products are derived using three main methods: remotely sensing, spatial interpolation, and reanalysis. These datasets encompass the major snow parameters—SWE, SD, and SCE. Like all gridded data, the utility of the snow data products is dictated by both their temporal and spatial dimensions, as well as the magnitude of bias in the estimates of snow parameter distribution. While local-scale analysis requires high to medium spatial resolution, regional and continental-scale analysis can be conducted using coarse-resolution data products. At this current juncture, coarse spatial resolution SWE (e.g., 4–25 km) time-series data products are prevalent, partly due to the challenges inherent in passive microwave, interpolation, and reanalysis techniques.

Snow depth is a crucial parameter for studying snow dynamics. Statistical interpolation methods have been employed to derive gridded SD for Finland and North America [39,42]. More recently, machine learning-driven methods were applied to fuse snow depth and the original gridded snow depth products, producing improved SD data [41]. SD data are one of the primary inputs that snow models assimilate to generate gridded SWE [39]. Despite their relevance as inputs to snow models, SD data generated through passive microwave techniques incur bias and underestimate SD, especially at over 100 cm depth [20]. For example, changes in passive microwave input and snow density parameters affect the quality of SWE data [43].

The Global Snow Monitoring for Climate Research’s (GlobSnow) SWE dataset is a typical example in which microwave remote sensing and synoptic weather station climate data are fused to derive SWE over a hemispherical scale [40,44]. GlobSnow SWE data incurs spatial discontinuity in mountainous regions. Consequently, research into developing snow data products for mountainous regions is an active area of scientific endeavour. Further, GlobSnow’s v3.0 resolution of 25 km × 25 km limits analysis at sub-kilometre scales. The European Space Agency’s Snow Climate Change Initiative (Snow_cci) data mitigates this challenge partially by improving SWE spatial resolution to approximately 11 km × 11 km [45].

Reanalysis, based on models for snow retrieval by assimilating ground weather stations data, is another type of gridded snow data product. Reanalysis data, however, tend to possess coarse spatial resolution and may come with profound bias as well as uncertainty in SWE variability [46]. Given the plethora of snow data products and their inherent variability in snow parameter representation, robust algorithms, and a combined analysis of the available datasets will likely yield informed insights into snow response to climate change and global warming.

### 2.2. Deep Learning Methods for Snow Modelling

Deep learning methods have been widely applied in diverse tasks, ranging from snow prediction to glacial/ice segmentation and change detection [47,48]. Siamese Attention Networks’ potential to detect in images has been demonstrated since the inception of the models. For example, a multi-attention model was deployed to detect changes as well as classify images [49]. In a related study, a multi-scale attention network was used for buildings and water segmentation [50]. A fusion-based attention network has also been shown to effectively detect change in bi-temporal imagery [51].

A variety of promising deep learning methods have been applied to study the cryosphere processes, especially snow, ice, and glacial dynamics across space and time. For instance, deep learning models have been used to elucidate the hydrology of snow-dominated watersheds in mountainous regions and related complex terrains [52]. Clouds and snow detection in images, an important task both in the context of data processing and snow modelling, is effectively accomplished using attention-based models [53,54]. The detection and characterization of glaciers and sea ice is yet another task in which attention-based mechanisms have been employed to improve model accuracy [47,55,56].

Over the past few decades, there has been an increased interest in the application of deep learning methods to enhance our understanding of the spatial and temporal variability of SWE. For example, machine-learning driven estimates of SWE across the western United States revealed a decline in snow parameters [57]. Similarly, daily SWE estimates in complex landscapes have been accomplished using deep learning models [48,58,59]. However, as demonstrated in [60], SWE prediction accuracy tends to vary remarkably across different models, thus highlighting the need for more robust architectures such as models with attention modules, as illustrated in [55,61].

## 3. Materials and Methods

**Figure 1:**
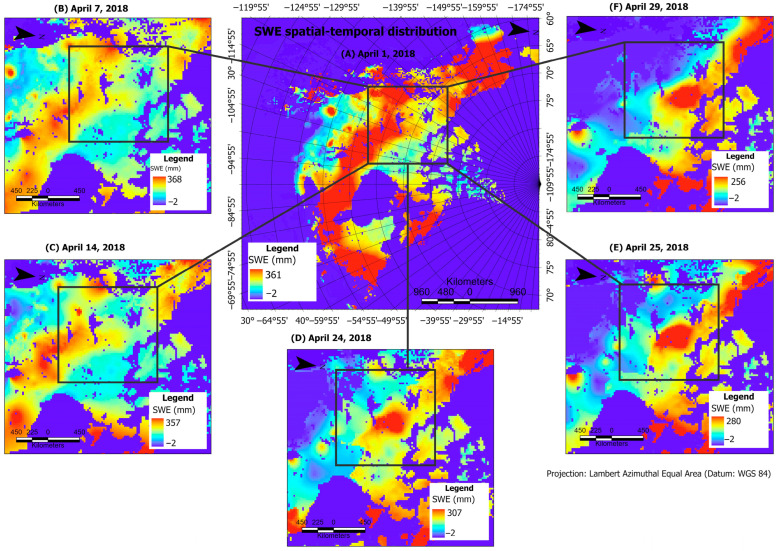
SWE spatial-temporal distribution across the Canadian cold regions in 2018. The insert maps (**A**–**F**) represent SWE distribution on 1, 7, 14, 24, 25, and 29 April, respectively.

Figure 1 depicts our study location in the cold regions of Canada. The provinces—Alberta, Yukon, and the Northwest Territories—cover a pronounced proportion of the study area. As can be observed from Figure 1, snow persists in the cold regions for longer days, beyond April, than in other areas of the Canadian land mass. Large-scale atmospheric processes occur in these regions, which result in temperature and water-related extremes. The climatology is characterized by the persistence of long days of near −0 °C temperatures and a mix of solid and liquid precipitation. These extreme climate-related events lead to winter storms, freezing rain, drought, spring floods, and wildfires [62,63,64]. We used the GlobSnow’s v3.0 SWE data, derived from the estimates of SWE using satellite-based passive microwave technology—Scanning Multichannel Microwave Radiometer, and Special Sensor Microwave/Imager (SSM/I) and Special Sensor Microwave Imager/Sounder (SSMIS) instruments. GlobSnow v3.0 incorporates bias-correction procedures that are implemented to account for uncertainties in the estimation of SWE. Additional details on this version of SWE data are provided in [37].

### 3.1. Training Sample Processing and Labelling

The SWE maps were dichotomized into “Change” (Negative Pairs) and “No Change” (Positive Pairs) instances. The SWE data that differed by one day were labelled as positive pairs (No Change), whereas the SWE maps that were two or more days apart were labelled as negative pairs (Change). Given that our training and validation data spanned five different locations and approximately 40 years, we adopted the structural similarity (SSIM) index to guide the labelling process with support from human expert supervision. The inherent spatial variability observed in the SWE maps and the lack of unique structures posed challenges to the SSIM index-guided labelling. More specifically, image pairs with SSIM values below 0.9 were easily detected by human experts to be different (Change instance), whereas SSIM values greater than 0.98 could not be easily distinguished and thus were labelled as similar (No Change instance). As noted in Malik and Robertson [31], SSIM index values between 0.9 and 0.98 were challenging to label as either No Change or Change instances. These samples were removed to avoid generating ambiguous labels. The removal of these samples resulted in 4% reduction in the training and validation datasets. Further details and guidelines on the deployment of the SSIM index for data labelling and the associated caveats are outlined in Malik and Robertson [31].

**Figure 2:**
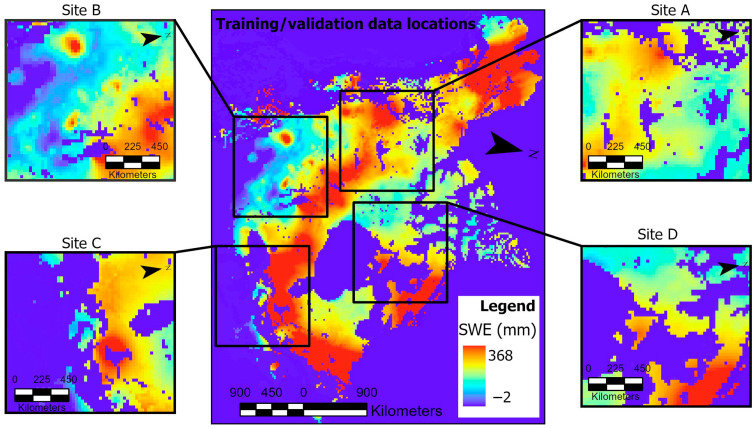
Training and independent validation data locations. Training and validation (Site A); independent validation only (Sites B–D).

We used SWE data from Site A for model development and Sites B, C, D for independent validation. For Site A, data from 1979 to 2001 and 2002 to 2018 were used for model development and independent validation, respectively. The training and validation data splits were 75% (training set) and 25% (validation set). The black bounding box in Figure 1 depicts the input data, consisting of 72 × 72 pixels. To test the generalization capability of the models, we employed data from different locations for independent validation (Figure 2, Sites B–D). Given that the data at Sites B–D were not used during model development, the independent validation data from these locations’ temporal span covered 1979 and 2018.

### 3.2. Siamese Attention U-Net Architecture

**Figure 3:**
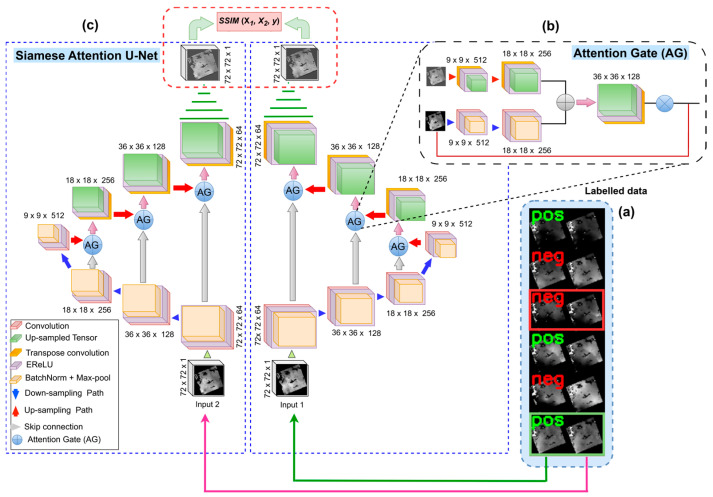
Siamese Attention U-Net architecture. A sample of labelled SWE data (**a**) consists of positive (pos) for similar pairs and negative (neg) for dissimilar pairs. The attention gate is depicted in (**b**) and the integration of SSIM index into the model’s architecture is shown in (**c**).

Our Si-Att-UNet with the attention module is depicted in Figure 3. While the Siamese network allows the model to learn shared feature representations from two input images, the U-Net with encoder–decoder architecture and skip connections simultaneously stimulates the learning of significant features for SWE representation and reconstruction. The encoder branch performs sequences of convolutions and down-sampling to generate the bottleneck features. The decoder branch then performs convolutional operations while up-sampling the bottleneck feature maps to reconstruct the original input image. The Attention module is a form of “spatial excitation”, which can potentially result in the extraction of significant features. We introduced the SSIM index to compute the similarity scores for the output SWE map pairs generated by the decoder branches (Figure 2, Site C) for comparison and parameter optimization using contrastive loss. This novel inclusion of the SSIM index in the U-Net model’s architecture was first proposed for change detection in [31].

Given the modified architecture, the Siamese Attention U-Net holds the potential to learn spatial structure encoding SWE variability and the underlying spatial processes (e.g., fraction of precipitation falling as snow and sub-zero temperatures) that generate SWE. However, the GlobSnow’s data spatial resolution is 25 km × 25 km; this requires a careful selection of the model’s filter parameter. Relating filter parameters to the field of view (FoV) of the neurons in the model’s first layer, the contextual window size equates to FoVs of 50 km × 50 km and 75 km × 75 km for the 2 × 2 and 3 × 3 filters, respectively. The spatial scale associated with the FoV increases in the higher layers of the network, resulting in the extraction of coarse resolution features. For daily change detection, a smaller filter parameter will likely capture the underlying local structure in SWE variability. Consequently, a 2 × 2 filter was adopted to effectively detect localized changes in SWE distribution. The hyperparameters adopted for model development include learning rate (0.01), batch size (100), and epochs (50). The stochastic gradient descent algorithm was adopted for training on an NVIDIA-enabled GPU with 12 GB of memory.

### 3.3. The SSIM Index and the Contrastive Loss Function

The expression for the SSIM index, proposed by Wang et al. [65], is given below.

(1) $$SSIM\left(x,y\right)=\frac{\left(2{µ}_{x}{µ}_{y}+C_{1}\right)\left(2\sigma_{xy}+C_{2}\right)}{\left({µ}_{x}^{2}{+µ}_{y}^{2}+C_{1}\right)\left(\sigma_{x}^{2}+\sigma_{y}^{2}+C_{2}\right)}$$

${µ}_{x}{\;and\;µ}_{y}$ are represent the mean of a block of pixels in image *x* and *y*, respectively; $\sigma_{x}^{2}$, $\sigma_{y}^{2}$ are, respectively, the variances of *x* and *y*; $\sigma_{xy}$ are *x* and *y* covariances; and $C_{1}{\;and\;C}_{2}$ are stabilizing constants. Wang et al. [65] provide the extensive mathematical details on the SSIM index.

The learning paradigm under the contrastive loss ensures that similar data pairs receive high scores while dissimilar data pairs receive lower scores. Consequently, the SSIM index value is maximized for positive (No Change) and minimized for negative (Change). The contrastive loss function, defined to operate with the SSIM index, is written as follows:

(2) $$L_{c}=(1-Y)\frac{1}{2}(s\left(x,y\right){)}^{2}+\left(Y\right)\frac{1}{2}\;{\left\{max(m-s\left(x,y\right),\;0)\right\}}^{2}$$

where $L_{c}$ denotes contrastive loss; m and s are margin and SSIM index, respectively; x,y denote a pair of SWE images; and Y represents labels. For positive pairs (No Change SWE), Y=1 (i.e., Y=1|{i=j}), and for negative pairs (Change SWE), Y=0 (i.e., Y=0|{i≠j}). We note that the margin parameter for the contrastive loss was set to unity (m=1) to align with the SSIM’s upper bound of 1. An extended mathematical illustration of how the SSIM index fits into the objective function’s optimization process is found in Malik and Robertson [31].

### 3.4. Deriving the Frequency of Daily Snow Water Equivalent Change Events

To estimate daily SWE change events, we first compute the daily similarity vector—SWEsim—as illustrated below:

(3) $$\vec{{\;{SWEsim}_{m}}_{\left\{d_{i}\;,\;d_{n}\right\}}\;}=model\;\left\{{SWE}_{m}\;\left({d_{i},\;d}_{i+1}\right)\right\}$$

where d and m represent day and month, respectively; $d_{i}$ denotes a SWE map observed on the ith day. The model term represents our Si-Att-UNet. We applied a 50% threshold to the resulting SWEsim values to derive the frequency of change events for each month. We adopted this threshold by examining the distribution of the model’s predictions of SWEsim on the independent validation data. At SWEsim values greater than or equal to 50%, the SWE map pairs were closely identical and could not be easily dichotomized by the human visual system. Thus, this threshold represents the “No Change” scenario, whereas values below this threshold were treated as change events.

### 3.5. SWE Change Point Detection

We utilized the changepoint package in R to compute change segments in each month. The package implementation follows the Prune Exact Linear Time (PELT) and Segment Neighbourhood Identification (SegNeigh) algorithms [66,67]. There are several adjustable parameters associated with PELT and SegNeigh, the choice of which influences their sensitivity to change point detection and the length (i.e., temporal dimension) of segments detected. The penalty parameter largely dictates the sensitivity of the algorithms to change segments in time-series data. Therefore, the PELT and SegNeigh methods were extensively explored with varying penalty terms for consistency and stability of change segments. We experimented with penalty parameters from 0.25 to 1.5 and found that the SegNeigh generated change points with reasonable temporal dimensions. The penalty parameter was adopted, and the values were set to 0.5 and 0.8 for mean and variance change segment computation, respectively. The PELT method consistently generated segments as short as one or two timestamps, regardless of the penalty magnitude.

## 4. Results

### 4.1. Model’s Accuracy Metrics

**Figure A1:**
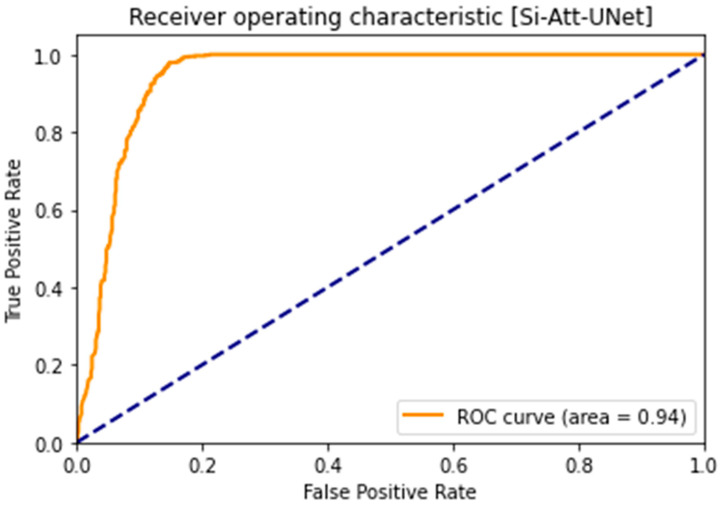
ROC-AUC for Siamese Attention U-Net.

The model’s accuracy on the prediction of SWE similarity and change was evaluated using the metrics—true positive rate (TPR), true negative rate (TNR), false positive rate (FPR), false negative rate (FNR), precision (PR), F1 score (F1), and overall accuracy (OA)—to assess the models’ prediction of SWE similarity and change. The F1 is preferred, especially when data exhibit class imbalance. The equations for the metrics are provided in Appendix C. The models’ accuracy from thresholds 40–50% is presented in Table 1 below. It can be observed that the F1-score increases as the confidence threshold parameter increases. The lowest F1 score (0.47%) was recorded at 40%; the model accurately detected all true positive samples. Contrarily, all the true negative samples were incorrectly detected as true positives. The Receiver Operating Characteristic Curve and Area Under the Curve (ROC-AUC) are depicted in Figure A1. The model’s ROC-AUC of 0.94 is within acceptable limits for the trade-off between TPR and FPR.

### 4.2. Ablation Studies

We conducted ablation studies to examine our model’s performance across different locations (Table 2). Given that the training data was obtained from Site A, the sample size (N) for independent validation was lower compared to the other locations (Sites B–D). It can be observed that the model’s F1 scores tended to be higher at Sites A and D. Contrarily, the F1 scores decreased marginally at Sites B and C.

Table 3 presents the accuracy metrics for CNN, U-Net base, and our model’s performance over a range of loss functions and similarity score combinations. The models’ performance was assessed using the Binary Cross-Entropy (BCE) and Contrastive Loss (CL) functions in combination with the Euclidean Distance (ECD) metric and the SSIM index. The models’ prediction of SWE change was conducted at Site B (Figure 2). All the models yielded varying F1 scores when the threshold parameter was altered. Nevertheless, the CL and SSIM index combination liberated an F1 score of ~98% at the lowest threshold (50%). We note that the U-Net base model also yielded a similar F1 score, but at a high threshold (75%). Without the SSIM index, the U-Net base and our model were less sensitive to true positive instances of SWE, yielding TPR scores of ~90% and ~83%, respectively.

**Figure 4:**
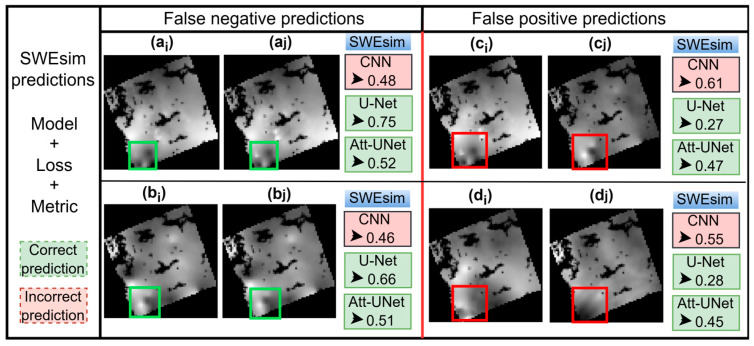
Models’ predictions on a sample of SWE data. False negative prediction pairs are (**a_i_**,**a_j_**) and (**b_i_**,**b_j_**), whereas false positive prediction pairs are (**c_i_**,**c_j_**) and (**d_i_**,**d_j_**). The false predictions are attributed to the CNN base model; the U-Net base and our Si-Att-UNet models’ predictions are correct based on the labelled data. Green and red rectangles are used to denote regions that are similar and regions that are different, respectively, in the underlying SWE data being predicted.

The CNN base, U-Net base, and our model’s predictions are depicted in Figure 4. The CNN base model’s false negative prediction pairs are (a_i_, a_j_) and (b_i_, b_j_) with SWEsim values of 0.48 and 0.46, respectively. The model’s false positive prediction pairs are (c_i_, c_j_) and (d_i_, d_j_) with corresponding SWEsim scores of 0.61 and 0.55. Based on the data labels, the U-Net and our model accurately predicted instances of No Change SWE, corresponding to Figure 4(a_i_,a_j_, b_i_,b_j_) and instances of SWE change, represented in Figure 4(c_i_,c_j_, d_i_,d_j_). We used green and red rectangles to demarcate regions that are similar and regions that are different in the SWE data, respectively.

### 4.3. Daily Snow Water Equivalent Change Events

Linear regression, Mann–Kendall (MK) trend test (Table 4),and change point analysis were performed to detect trends in the daily SWE change events from 1979 to 2018. The statistics, *S*, *tau*, and *p-value* are derived from the MK test, while the *R^2^* is estimated using linear regression. It can be observed that in January and February there were no statistically significant trends in the frequency of change events. Conversely, a significant increase in the number of change events occurred in March and April. However, SWE change events exhibited a profound increase in April (*tau* = 0.38, *p-value* < 0.05) than those observed in March. Figure A3 illuminates the distribution of SWE change events from 1979 to 2018. April portrayed a high frequency of change events, whereas January depicts high variability.

**Figure 5:**
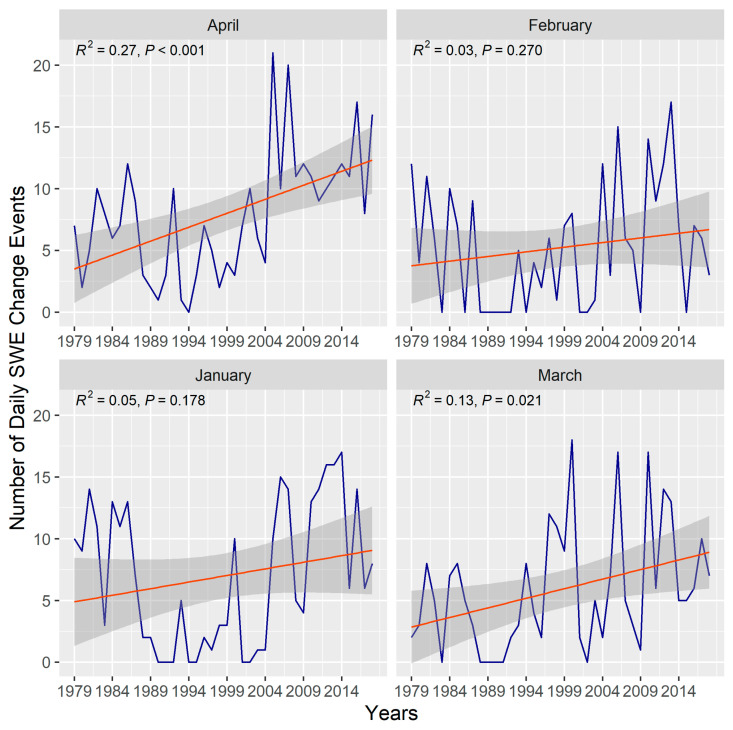
Frequency of daily SWE change events. The orange line is derived from linear regression conditioned on the frequency of change events over time. The grey is the 95% confidence interval.

Figure 5 presents the daily count of SWE change events from 1979 to 2018. The change events were more frequent in April than in January, February, and March. Between 1979 and 1987, the change events were relatively low. The frequency of change events further declined substantially between 1988 and 1993. Contrarily, the change events consistently increased after 1994. April recorded the highest amount of non-zero change events (one zero change in 1994). The maximum number of change events (i.e., 20–21) occurred in April between 2005 and 2007. January, February, and March recorded non-zero change events for two to three consecutive years.

### 4.4. Daily SWE Change Segments

**Figure 6:**
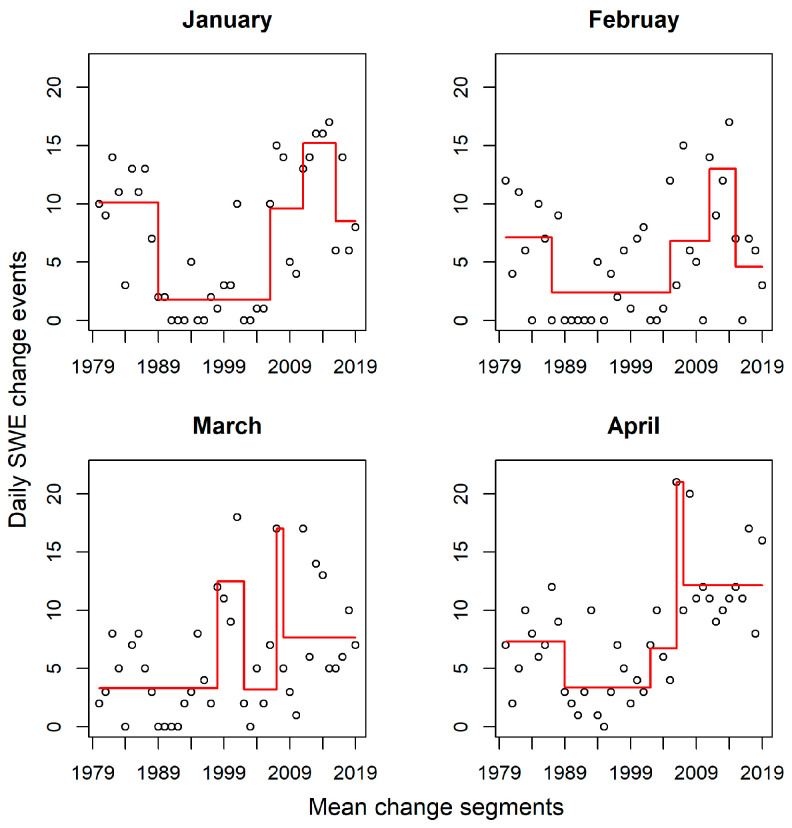
Mean change points for daily SWE change events. Change segments are represented using red lines. The horizontal width of the lines demarcates change segments.

Figure 6 presents the mean change segments for the change events in the four months. Each segment’s length is the width of the horizontal lines, while the vertically oriented lines demarcate the end of one segment and the beginning of another. The change point algorithm consistently detected change segments from 1979 to 1987 in all the months. However, March depicts a longer change segment spanning 1979 to 1999. Again, a relatively longer segment (2009 to 2018) was detected in March and April, yet the mean change point was higher in April (~12 days) than in March (~9 days). It is also interesting to note that a major segment was detected in January, February, and April, stretching from 1989 to 2004. Conversely, this segment appeared fragmented into two minor change points in March.

### 4.5. Temperature Anomaly

**Figure 7:**
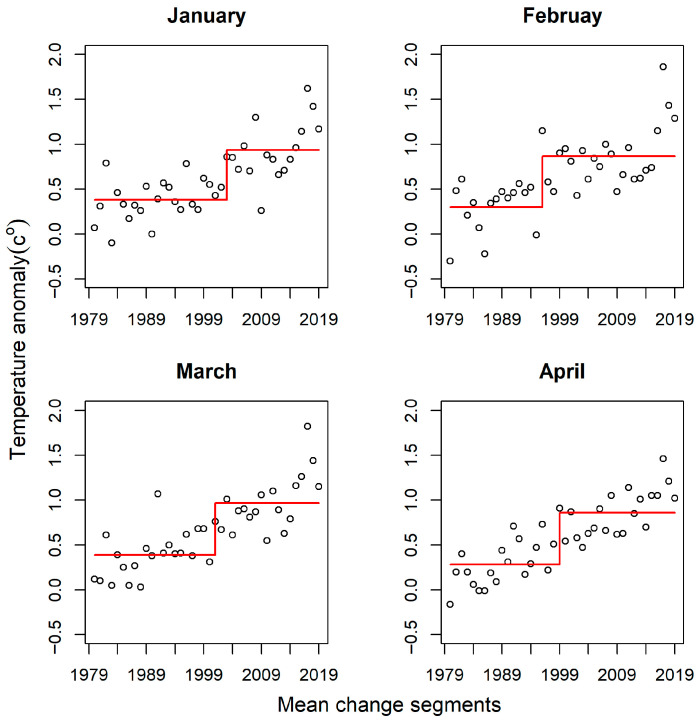
Northern hemisphere temperature anomaly mean change segments. The circles represent temperature anomalies, while the red lines denote the mean change segments.

Figure 7 presents NOAA’s Land and Ocean surface temperature anomalies over the northern hemisphere from 1979 to 2018. The red line denotes change segments detected by the change-point algorithm, whereas the circles are monthly temperature anomalies. For February, the temperature anomaly remained low from 1979 to 1994. This temporal window was detected as a mean change segment. Temperature anomalies remained relatively low in the earlier decades (i.e., 1979–1999) for the rest of the months. Conversely, the anomalies increased dramatically in the subsequent decades. This rise in anomaly was detected as the second change point in the temperature anomaly time-series. The highest anomaly readings were observed after 2015 for all the months. Sub-zero temperature anomalies occurred in all the months except March. Overall, there were two consistent major change segments for all the months; one occurring in the earlier decades (1979–1999) and another in the latter decades (2000–2018).

### 4.6. Precipitation Anomaly

**Figure 8:**
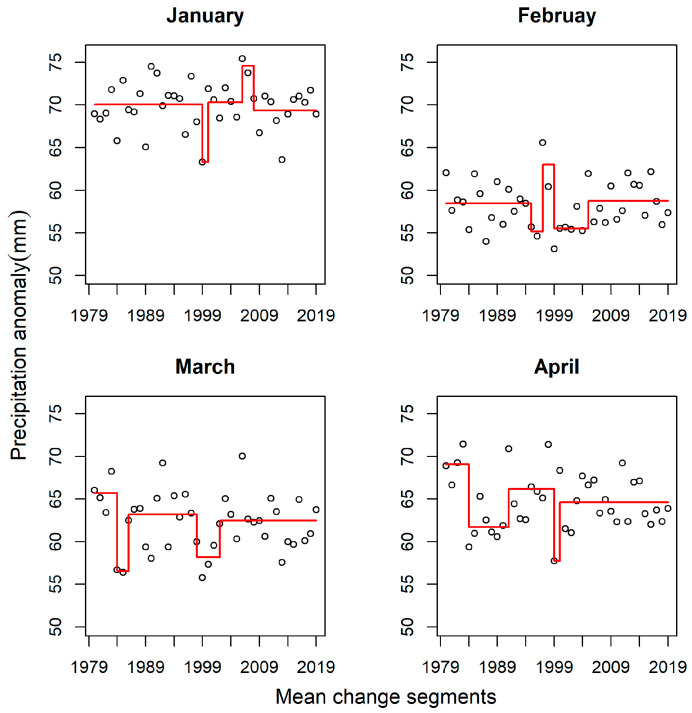
Northern hemisphere precipitation anomaly mean change segments. Red lines are used to change segments, while the circles denote precipitation anomalies.

Figure 8 depicts the mean change points derived from NOAA’s Land and Ocean precipitation anomaly data. There were three major change segments in January and February, and four in March and April. The change points and segments for January and February exhibited a consistent pattern for the first 1.5 decades (1979–1994) as well as the last decade (2009–2018). However, it is worth emphasizing that the mean of the change segments was higher in January (i.e., 70 mm) than in February (i.e., 58 mm). March and April also elicited similarities in the pattern of mean change segments. For example, the lengths of the first change segment between 1979 and 1984 were approximately congruent. The mean of the segments varies marginally, reaching a maximum of 66 mm and 68 mm for March and April, respectively. The last mean change segments in March and April span a similar temporal window (i.e., 2000–2018) with nearly identical mean values (~63 mm). A major change segment was observed in March between 1985 and 1999. In a similar vein, between 1985 and 1990, a major change segment (mean of ~62 mm) was detected in April. Additionally, a change segment occurred between 1990 and 1999. It is worth noting that the last decade (2008–2018), incurred major change segments for all the months.

## 5. Discussion

**Figure A2:**
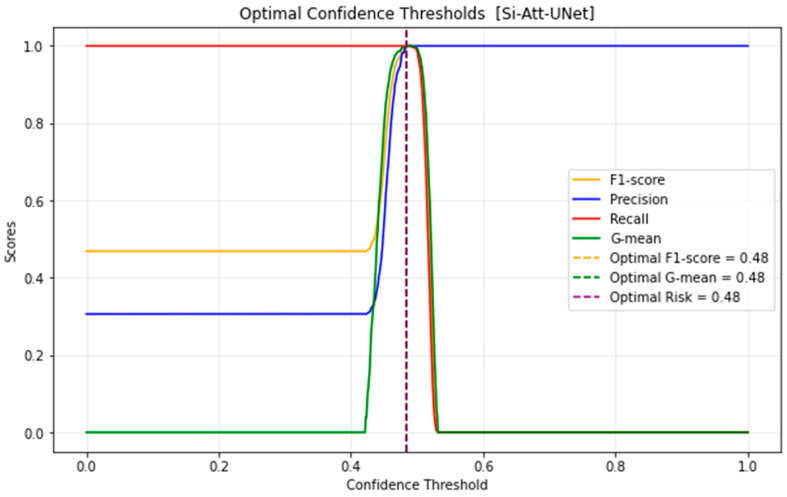
Optimal confidence thresholds for SWE change detection. The optimal geometric mean (G-mean), F1-score, and the risk of misclassification converge at 0.48 threshold.

**Figure A3:**
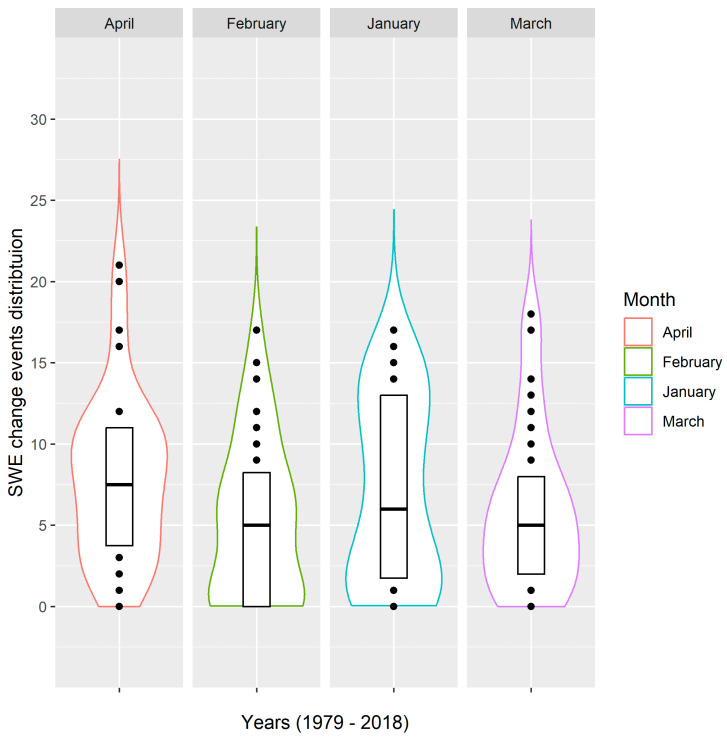
SWE change events distribution from 1979 to 2018.

The daily SWE change events detection task was cast as a computer vision problem in which a pre-trained Si-Att-UNet model was deployed to compare a pair of SWE maps sampled at two temporal windows—the current time and future time. The SWEsim values were flagged as “Change” or “No Change” instances if a threshold value was exceeded. Change events were detected using a threshold of 50% daily SWE change events. As shown in Table 1, except for TPR, which remained 100%, all the other accuracy metrics varied as the threshold parameter was altered from 40% to 48%. Figure A2 presents a visualization of the accuracy metrics as the threshold changes. There was a trade-off between FNR and FPR, whereby increasing the threshold beyond 50% resulted in an elevated FPR or reduced TPR while increasing TNR. This behaviour of the model implies that No Change instances of SWE are easy to detect, even at very low threshold parameters. The opposite is true for the Change instances; thus, a higher threshold parameter was required to detect dissimilar SWE. This highlights the challenging nature of change detection problems. Although the number of FPR began to increase as the threshold increased, the 50% threshold appeared to be the optimum value for maximizing the model’s accuracy (F1-score). We note that the model’s prediction accuracy (F1 score) decreased marginally to ~97% at Sites B and C but remained high at Site D (Table 2). Thus, the model possesses the potential to generalize to unseen data in different regions without a significant reduction in its accuracy.

The CNN base, U-Net, and our Si-Att-UNet accuracy varied over a range of loss functions and similarity metrics combinations (Table 3). The models’ optimum threshold parameter for accurate detection of “Change” and “No Change” instances of SWE also tended to vary with loss functions and similarity metrics combinations. The combination of CL and SSIM index yielded the highest accuracy, 98% and 97%, respectively, for the U-Net base and our model. However, it turns out that the U-Net requires a high threshold parameter to accurately detect change, especially, dichotomizing true negative instances of SWE. This suggests that our model is more sensitive to changes in SWE than the U-Net. As shown in Figure 4, both the U-Net and our model’s predictions were correct. However, SWEsim scores differed; while the U-Net assigned high scores to No Change pairs, and low scores to Change pairs, the Si-Att-UNet scores are elevated yet within the optimal threshold (i.e., 0.48) (Figure A2) for best performance. It is important to reiterate that the U-Net similarity attribution tended to be more intuitive and interpretable than our model (Figure 4).

Mann–Kendall trend test and linear regression were performed on the threshold values to estimate the significance in the direction and magnitude of daily change events as in [14,68]. Daily SWE change events trends in April (*tau* = 0.38) and March (*tau* = 0.25) exhibited a statistically significant increase from 1979 to 2018. January and February showed no significant trend in the frequency of change events. The relatively high frequency of change events in April is indicative of pronounced daily variability in snow distribution and melt-off patterns. This characteristic daily melt-off is obvious in the April 2018 SWE maps depicted in Figure 1. As can be observed from the figure, the SWE melt-off pattern is more pronounced from April 24 to 29. The *tau* value of 0.38 (Table 2) supports an increasing snow melt-off pattern in April. The daily melt-off is predominantly a function of changes in temperature and precipitation [17,69]. Given that the frequency of change events was high in April and increased over time, it can be postulated that climate-forcing agents significantly altered the snow regime in April. However, we emphasize that a daily localized comparison of temperature and precipitation over the study area is required to substantiate this conclusion. The SegNeigh change-point algorithm revealed consistent change segments in the earlier period (i.e., 1979–1987) across all months and years. Shorter segments are indicative of the varying influence of the effects of the climate drivers (i.e., temperature and precipitation) [70,71,72]. The mean of the segments tended to occur at higher change event frequencies in January, February, and April; this suggests a high variability in daily snow deposition processes (e.g., low temperature and high fraction of precipitation falling as snow), particularly in January and February.

The pattern observed in April SWE distribution is related to a consistent decline in snow parameters over the years. The last decade of April (i.e., 2008–2018) exhibited the highest frequency of change events. This period recorded the longest mean change segment among all the months. Daily SWE melting process evolves more rapidly in April, resulting in low SWEsim values, and thus increasing the frequency of daily change events (Figure A1). Contrarily, the mean change segments for March were associated with lower change event counts, especially in the first two decades (i.e., 1979–1999). Snow parameters (e.g., snow depth, snow density, and snow cover extent) tend to reach their peak in March [28]; this implies the snow parameters were highly stable in March, resulting in higher SWEsim values and, consequently, a low frequency of change events.

The NOAA’s Land and Ocean Surface temperature and precipitation anomaly at a monthly temporal resolution over the northern hemisphere holds the potential to elucidate the effects of climate-forcing variables on the snow regime and related cryosphere processes. Figure 5 and Figure 6 present average monthly temperature and precipitation mean change points over 40 years. The temperature anomaly trends depicted an inverse relationship with interannual SWE change events. As quantitatively derived from the NOAA data, the temperature anomalies increased by +0.25 °C/decade (January), +0.26 °C/decade (February), and +0.28 °C/decade (March and April) [73]. Temperature anomalies were lower in the earlier decades (i.e., 1979–1987); this coincides with the fewer change events detected in this temporal window. The latter years (1990–2018) were characterized by high temperature anomalies; again, this concurs with the high frequency of daily change events observed after 1990.

The trends in average monthly precipitation show variable patterns over the northern hemisphere. Precipitation anomalies increased marginally in January (+0.01 mm/decade) and February (+0.02 mm/decade). March and April recorded a significant reduction in precipitation anomalies, corresponding to −0.32 mm per decade and −0.3 mm/decade, respectively [73]. Although the relationship between precipitation and the frequency of change events was not entirely consistent, the mean change segments offer insights into the variation in the snow-related change events in each month. For instance, February recorded the highest number of zero-change events, which is in consonance with the observed low precipitation anomalies. Elevated precipitation anomalies were detected in January, with the corresponding high number of change events. Similarly, high anomalies of precipitation were observed in March and April, resulting in an equal number of major mean change segments. Despite the realization of closely similar precipitation anomaly patterns in March and April, there were no non-zero change events in April. Zero-change events are positive signs of a stable snow regime and cryosphere response to climate change and global warming; therefore, the snow regime in April may have been largely influenced by climate drivers (e.g., temperature and precipitation). We note that due to the mismatch between the GlobSnow’s SWE and NOAA data temporal resolution, no correlation test was performed to determine the response of SWE to temperature and precipitation; as a result, their relationship is hypothesized but not causality attributed to climate variables. Nonetheless, a significant decline of snowpack, largely driven by warming trends, has been recorded in the Western parts of the United States during April [74].

It is important to emphasize that temperature and precipitation exerted variable effects on snow in the study area. This stems from the fact that the two spatial processes operate at different spatial scales and may influence snow accumulation and melting processes differently [21,75]. Furthermore, precipitation can be decomposed into the fraction falling as snow and the fraction falling as rain [76]. While the fraction falling as snow potentially results in increased snow accumulation, the fraction falling as rain reduces snowfall and could further deteriorate ground snow conditions. A combination of these scenarios can influence the frequency of change events. For instance, SWE variability has been shown to depend on variations in snowfall and ground snow fractions than on total precipitation [30].

## 6. Conclusions

Our Siamese Attention U-Net model detected daily snow-related change events in the winter season using SWE, a parameter of snow. At low threshold parameters, the model effectively detected instances of No Change (similar) SWE pairs; however, detecting Change (dissimilar) SWE map pairs was challenging, requiring higher threshold parameters to increase the TNR. The U-Net base model with SSIM index and CL was found to be competitive with our model, but less sensitive to changes in SWE, requiring a higher threshold parameter. This suggests that the models may be used complementarily for change detection. The U-Net and the Si-Att-UNet have the potential to be deployed for snow monitoring on edge devices; however, for mobile devices with limited memory, post-training quantization may be required, especially to reduce the size of our model. Furthermore, our implementation of the model frameworks utilizes Tensorflow, Keras, Scikit-learn, and geospatial data abstraction libraries. These open-source libraries can be integrated into Artificial Intelligence (AI) platforms, such as Azure OpenAI services, Google AutoML, and Amazon SageMaker, to aid in full-stack and No-Code model development and deployment. However, setting up backend server and database services may require Node.js, MongoDB, and GeoDjango.

Daily SWE change events were high in the first decade after 1979 (i.e., 1979–1987). Contrarily, the daily change events declined between 1989 and 2004 to a range of two to three events. This temporal window would be suitable for selection as the base or reference year for analysis geared toward daily SWE change detection. Given the low frequency of change events, SWE data between 1979 and 1987 may be selected as the reference year for interannual change detection, but care should be exercised due to the temporal discontinuity in this temporal window. While precipitation anomalies were found to influence the frequency of change events, low temperatures were the primary driver of low SWE change event frequencies in the first 1.5 decades after 1979 (i.e., 1979–1994). Temperature anomalies increased exponentially after this period, resulting in a high frequency of change events. Overall, the daily snow-related change events were significant in March and April; however, April exhibited the highest frequency of change events than the other winter months; this pattern is indicative of the dramatic consequences of the changing climate regime on snow dynamics and cryosphere processes.

We note that our analysis has inherent limitations: (a) The GlobSnow SWE data and the NOAA climate variables. As mentioned previously, the GlobSnow SWE data were sampled every other day from 1979 to 1987. The temporal discontinuity may have magnified the variability between SWE maps, causing lower SWEsim values to dominate, biassing the results in favour of high frequency of daily change events. Therefore, the analysis of complete daily snow data within this time window would provide a more reliable estimate of the number of change events. Furthermore, unlike SCE, the retrieval of SWE is more challenging and comes with a variety of observational uncertainties [26]. For example, the input passive microwave and synoptic weather station data may alter SWE spatial and temporal variability across different products [20]. Therefore, comparing the model’s performance across different SWE data products would yield additional insight into daily SWE changes, as this would help account for the influence of bias in SWE data. Furthermore, conducting change event analysis with high or medium spatial resolution SWE data will likely unravel the effects of climate regime change on snow dynamics, especially at local scales. The NOAA climate data represent monthly average temperature and precipitation anomalies over the northern hemisphere. The analysis of daily climate variables across our study area is likely to yield a more meaningful correlation between change events and climate effects. (b) The Si-Att-UNet model’s output SWEsim values’ threshold potential bias. Although the threshold was selected based on the value that produced optimum model performance, there was a trade-off between the model’s performance on TPR and FNR at varying thresholds. Additionally, as shown in Table 3, the models’ accuracy at inference varied significantly with changing threshold parameters. Thus, a statistically robust method (e.g., Cohen’s Kappa) and experts’ views could help us arrive at a more reliable threshold value.

## Figures and Tables

**Table 1:** Siamese model’s prediction accuracy.

Threshold	TPR	FPR	TNR	FNR	PR	F1	OA
40%	100.00	100.00	0.00	0.00	30.60	0.47	30.60
45%	100.00	37.04	62.94	0.00	54.34	70.0	74.28
46%	100.00	12.56	87.44	0.00	77.84	88.0	91.29
47%	100.00	3.52	96.48	0.00	92.60	96.00	97.56
48%	100.00	0.77	99.23	0.00	98.29	99.00	99.47
50%	98.61	0.00	100.00	1.39	100.00	99.30	99.57

**Table 2:** Siamese model’s prediction accuracy over different locations.

Location	TPR	FPR	TNR	FNR	PR	F1	N
Site A	96.61	0.00	100.00	1.39	100.00	99.30	1882
Site B	100.00	0.52	99.48	0.00	95.56	97.73	5500
Site C	97.81	0.60	99.40	2.29	97.10	97.45	5169
Site D	100.00	0.00	100.00	0.00	100.00	100.00	7410

**Table 3:** Siamese models comparison with varying loss functions and similarity metrics.

Model Architecture	Loss Functions		Similarity Metrics		Accuracy Metrics				
	BCE	CL	ECD	SSIM	TPR	TNR	PR	F1	Threshold
CNN base	Yes	No	Yes	No	92.84	99.44	94.86	93.84	70%
U-Net base	Yes	No	Yes	No	89.56	97.12	77.55	83.12	65%
U-Net base	Yes	No	No	Yes	100.00	98.37	87.18	93.15	82%
U-Net base	No	Yes	No	Yes	96.71	99.96	99.60	98.14	75%
Si-Att-UNet	Yes	No	Yes	No	83.17	98.50	86.00	84.56	85%
Si-Att-UNet	Yes	No	No	Yes	100.00	99.14	92.82	96.28	70%
Si-Att-UNet	No	Yes	No	Yes	100.00	99.48	95.56	97.73	50%

**Table 4:** Mann–Kendall test and linear regression statistics.

Month	S	tau	*p*-Value	R^2^
January	7291	0.16	1.4 × 10^−1^	5.0 × 10^−2^
February	7128	0.13	2.7 × 10^−1^	3.0 × 10^−2^
March	7275	0.25	2.6 × 10^−2^	1.3 × 10^−1^
April	7310	0.38	7.9 × 10^−4^	2.7 × 10^−1^

## Data Availability

The data used to train the model are available at https://www.globsnow.info/swe/archive_v3.0/ (accessed on 1 December 2024). The authors also plan to make the sample data and the trained model available after the manuscript is accepted or upon request. The NOAA climate anomaly data are also available at https://www.ncei.noaa.gov/access/monitoring/climate-at-a-glance/global/time-series (accessed on 8 March 2025).

## Appendix C

(A1)
$$Recall\;\left(TPR\right)=\frac{TP}{TP+FN}$$

(A2)
$$Precision=\frac{TP}{TP+FP}$$

(A3)
$$F1\;score=\frac{2TP}{2TP+FP+FN}$$

(A4)
$$F1\;score=\frac{2TP}{2TP+FP+FN}$$

(A5)
$$OA=\frac{TP+TN\;}{TP+TN+FP+FN}$$
